# Real-Time PCR Quantification of 87 miRNAs from Cerebrospinal Fluid: miRNA Dynamics and Association with Extracellular Vesicles after Severe Traumatic Brain Injury

**DOI:** 10.3390/ijms24054751

**Published:** 2023-03-01

**Authors:** Lara Valenčić Seršić, Vedrana Krušić Alić, Maša Biberić, Siniša Zrna, Tin Jagoić, Janja Tarčuković, Kristina Grabušić

**Affiliations:** 1 Department of Anaesthesiology, Resuscitation, Emergency and Intensive Care Medicine, Faculty of Medicine, University of Rijeka, 51000 Rijeka, Croatia; 2 Anaesthesiology, Intensive Medicine and Pain Treatment Clinic, Clinical Hospital Centre Rijeka, 51000 Rijeka, Croatia; 3 Department of Physiology, Immunology and Pathophysiology, Faculty of Medicine, University of Rijeka, 51000 Rijeka, Croatia; 4 General Hospital Pula, 52100 Pula, Croatia

**Keywords:** Traumatic Brain Injury, Cerebrospinal Fluid, MicroRNAs, Size Exclusion Chromatography, Extracellular Vesicles, CD81 protein

## Abstract

Severe traumatic brain injury (sTBI) is an intracranial damage triggered by external force, most commonly due to falls and traffic accidents. The initial brain injury can progress into a secondary injury involving numerous pathophysiological processes. The resulting sTBI dynamics makes the treatment challenging and prompts the improved understanding of underlying intracranial processes. Here, we analysed how extracellular microRNAs (miRNAs) are affected by sTBI. We collected thirty-five cerebrospinal fluids (CSF) from five sTBI patients during twelve days (d) after the injury and combined them into d1–2, d3–4, d5–6 and d7–12 CSF pools. After miRNA isolation and cDNA synthesis with added quantification spike-ins, we applied a real-time PCR-array targeting 87 miRNAs. We detected all of the targeted miRNAs, with totals ranging from several nanograms to less than a femtogram, with the highest levels found at d1–2 followed by decreasing levels in later CSF pools. The most abundant miRNAs were miR-451a, miR-16-5p, miR-144-3p, miR-20a-5p, let-7b-5p, miR-15a-5p, and miR-21-5p. After separating CSF by size-exclusion chromatography, most miRNAs were associated with free proteins, while miR-142-3p, miR-204-5p, and miR-223-3p were identified as the cargo of CD81-enriched extracellular vesicles, as characterised by immunodetection and tunable resistive pulse sensing. Our results indicate that miRNAs might be informative about both brain tissue damage and recovery after sTBI.

## 1. Introduction

Traumatic brain injury (TBI) is a disruption of brain anatomy and function due to the action of an external force [1]. This may happen in different conditions, such as the head striking an object (blunt trauma), the brain undergoing a rapid acceleration/deceleration movement, or a foreign body penetrating the brain [2]. The two leading causes of TBI are falls and motor vehicle accidents. These injury mechanisms result in the highest incidences among specific age groups: falls are most prominent in early childhood (0–4 years) and older adults (>65 years), while adolescents and young adults (15 to 24 years) are mostly affected by motor vehicle accidents [3].

In terms of pathogenesis, TBI involves a complex process that starts as a primary injury at the moment of physical impact to the head, and continues as a secondary injury during hours, days or even weeks after the primary injury [3]. The secondary injury is a result of different biochemical and cellular events triggered by the primary injury. They include excitotoxicity, mitochondrial disfunction, oxidative stress, lipid peroxidation, neuroinflammation, blood brain barrier disruption, axon degeneration, and apoptotic cell death [4].

In clinical settings, TBI is classified as mild, moderate or severe. The initial assessment is based on the international Glasgow Coma Scale (GCS), with predefined points for eye, verbal and motor response. The scale ranges from 3 to 15 points, where 3 points are assigned to a comatose patient with no response, and 15 are assigned to a fully alert and cooperative patient. Taken together, GCS subranges of 3–8, 9–12 and 13–15 define the severe, moderate and mild TBI, respectively [5]. Before the final diagnosis is made, additional diagnostic methods, including neuroradiological imaging and neurological examination, are used to complement the GCS-based clinical profile of the patient. Next to intensive treatment measures, severe TBI (sTBI) can involve emergent surgical interventions including decompressive craniectomy and/or placing a catheter into cerebral ventricles; or a system called an external ventricular drain (EVD) to monitor and control the intracranial pressure (ICP) values [6]. Even when acute treatment is successful, long term survivors from sTBI have an increased risk of mortality and disability due to the acquired central nervous system (CNS) damage [7,8].

Despite the significant progress in the field of TBI, both in terms of pathophysiology as well as diagnostic and treatment procedures, several intrinsic TBI features are still associated with challenges in clinical settings and require a deeper understanding of processes in the brain after the injury. Not only can the clinical presentation of TBI be very different, but it can also change rapidly due to the dynamic nature of the secondary injury. On top of that, continuous monitoring of brain morphological changes via neuroradiological imaging is not applicable for both practical and safety issues. Therefore, additional diagnostic and prognostic tools for evaluating the brain function after TBI are greatly needed. Recent studies identified circulating biomarkers in body fluids such as cerebrospinal fluid (CSF) and blood with growing interest in microRNAs (miRNAs) [9,10].

miRNAs are small (19–28 nucleotides), non-coding RNAs involved in posttranscriptional gene regulation. This is especially noticeable in CNS, since approximately 70% of known miRNAs are estimated to be expressed in nervous tissue, both in physiological and pathophysiological conditions [11]. Similarly, TBI has been reported to alter different miRNAs [9]. The downregulation of plasma miR-16 and miR-92 and the upregulation of plasma miR-765 have been detected within the first 24 h after sTBI [12]. Increased plasma miR-92a and miR-16 were found in mild TBI patients [12]. A post-mortem gene expression analysis of the human cerebellum after sTBI detected 13 miRNAs, but found no clinical correlation [13]. Elevated serum miR-93, miR-191, miR-499, miR-21 and miR-335 were noticed in sTBI patients and were suggested as biomarkers for the TBI detection and progression [14,15]. Furthermore, miR-124, miR-219a, miR-9, miR-137, and miR-128 might be more CNS-specific, as determined by a bioinformatic analysis [16]. Serum levels of miR-219a-5p have been indicated as a potential indicator for the diagnosis and prognosis of sTBI while six other miRNAs, including miR-103a-3p, miR-302d-3p, miR-422a, miR-518f-3p, miR-520d-3p and miR-627, were significantly upregulated in both severe and mild TBI patients in comparison to healthy controls [17]. Another studies of plasma and CSF samples also identified a panel of miRNAs as a potential signature specific of TBI [18,19]. While the search for miRNA biomarkers in CSF and blood/plasma serum brings the advantage of analysing the body fluid which is in direct contact with the brain or is easily accessible, salivary samples have also been utilised to characterise miRNA after TBI, as recently reviewed [20]. Taken together, the majority of researches used serum and plasma samples as the main investigated biofluids in TBI patients. The resulting miRNA species with potential diagnostic and prognostic value varied, which could be due to differences in methodology in sampling and analyses.

An additional feature of extracellular miRNAs, which might affect isolation and characterisation, is that miRNAs can circulate either bound to proteins or as the cargo of extracellular vesicles (EVs) [21]. EVs are membrane-enveloped nanostructures (20–1000 nm in diameter) that are secreted by cells into body fluids. They carry their diverse content including lipids, proteins and nucleic acids, such as miRNAs [22]. EVs are very heterogenous in their size and molecular cargo, but their content is believed to reflect the type and current state of the originating cells [23]. Since EV content is cell-specific and EVs are accessible from body fluids, EVs have a biomarker potential which is especially valuable for organs with no or difficult access, such as the brain, after sTBI [24]. EVs in CSF after sTBI were shown to change their number, size and protein composition, as well as to carry miRNA resulting in considering EV-miRNAs as biomarkers for TBI [25,26,27]. EV miRNAs isolated from human and mouse plasma samples also showed potential in TBI classification: a panel of eight miRNAs (miR-150-5p, miR-669c-5p, miR-488-3p, miR-22-5p, miR-9-5p, miR-6236, miR-219a.2-3p, miR-351-3p) were identified in a mouse TBI model, and four miRNAs (miR-203b-5p, miR-203a-3p, miR-206, and miR-185-5p) were differentially regulated in TBI patients when compared to healthy controls, respectively [28]. Taken together, various miRNAs from different body specimens were shown to change their levels after TBI, whereby some of these changes might be in the context of EVs.

To provide a more systematic approach, we decided to monitor a set of miRNAs in several time points after sTBI. We collected CSF samples during the 12 days (d) after sTBI and combined them into d1–2, d3–4, d5–6 and d7–12 CSF pools. We quantified 87 miRNAs by real-time PCR in all CSF pools, as well as in EV- and free protein (FP)-fractions obtained after size exclusion chromatography (SEC). We found that the majority of targeted miRNAs showed the highest levels in d1–2 and d3–4, which were also compromised by haemolysis. Furthermore, most miRNAs were associated with FPs. Although the main limitation of the study is the low number of patients and CSF samples, our data show a high range of miRNA concentrations and identify miRNA candidates indicative for ongoing processes in the brain.

## 2. Results

### 2.1. Patients with Severe Traumatic Brain Injury and Four Pools of Consecutive CSF Samples from the Acute Phase of Treatment

Severe traumatic brain injury (sTBI) changes the molecular composition of cerebrospinal fluid (CSF) due to the very nature of intracranial tissue damage [25,29]. To enable better insight into CSF molecular dynamics, we included sTBI patients whose acute treatment required intracranial pressur (ICP) monitoring and management by placing the external ventricular drain (EVD) soon after the primary injury. CSF samples were obtained from 5 sTBI patients, four males and one female, with ages ranging from 19 to 49 years and a mean age of 33.8 ± 12.8 years (Table 1). All but one patient had a favourable outcome three months after discharge as defined by a Glasgow Outcome Scale (GOS) score of 4 or 5.

**Figure 1:**
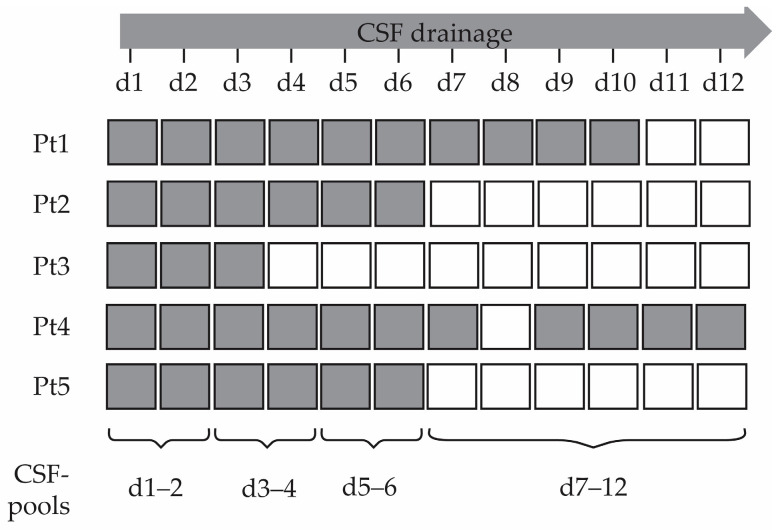
**Collected individual cerebrospinal fluid (CSF) samples and the creation of consecutive CSF pools from severe traumatic brain injury patients.** Individual CSF samples from five patients (Pt) were collected on indicated (grey shaded boxes) days (d) and combined into four consecutive CSF pools (d1–2, d3–4, d5–6 and d7–12), which were used for downstream analyses.

A total of thirty-five individual CSF samples were collected daily for up to twelve days after the injury until EVD was in place, a decision which was at the discretion of the supervising physician (Figure 1). To facilitate downstream analyses, we combined consecutive CSF samples into four pools: days 1 and 2 (d1–2), d3–4, d5–6 and d7–12. The d1–2 and d3–4 were derived from five sTBI patients, while d5–6 and d7–12 CSF pools were derived from four and two sTBI patients, respectively. However, all CSF pools contained between eight and ten individual CSF samples, resulting in comparable CSF mixtures regarding the sample number and volume. The first three CSF pools contained samples from both patients with good (GOS 4–5) and fatal (GOS 1) outcomes, while the latest CSF pool was comprised of CSF from patients with good outcomes (Table 1).

### 2.2. Cerebrospinal Fluid after sTBI Contains miRNA during the First 12 Days after the Injury but Is Also Compromised by Haemolysis

**Figure 2:**
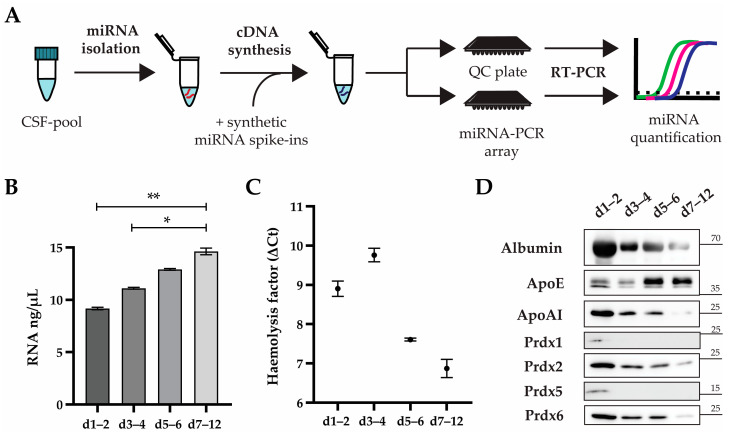
**microRNA (miRNA) is detected in all four cerebrospinal fluid (CSF) pools along with haemolysis and plasma proteins.** (**A**) The study overview comprising miRNA isolation, addition of miRNA spike-ins before cDNA synthesis, and miRNA quantification by real-time PCR, with predesigned arrays containing primers for 87 target miRNAs (miRNA-PCR array) or miRNAs for a quality control (QC) plate. (**B**) miRNA was isolated from the CSF pool of indicated days (d) after severe traumatic injury, and RNA concentration was determined spectrophotometrically. The mean values of three miRNA isolations with standard deviations are shown. * *p* < 0.05 and ** *p* < 0.01 by one-way nested ANOVA analysis with Tukey’s post hoc test for multiple comparisons. (**C**) Haemolysis factor as determined by real-time PCR with cDNAs from indicated CSF pools applied to amplify haemolysis dependent and independent miRNAs on a QC plate. The mean values of three experiments with standard deviations are shown. (**D**) The immunodetection of depicted proteins in specified CSF pools after western blot. Protein sizes are designated in kilodaltons. Representative images are shown.

Several studies have shown that diverse microRNAs (miRNAs) can be detected in human CSF after sTBI, but no comprehensive comparison of miRNA types and levels during patient recovery have been described. To provide a more systematic analysis of miRNA, we decided to simultaneously quantify a set of 87 miRNAs in the consecutive CSF pools spanning the twelve days after the injury. We applied a commercially available PCR-array containing primers for CSF-associated miRNAs and enabling absolute miRNA quantification based on spike-in miRNAs (Figure 2A).

We isolated miRNAs from four CSF pools and determined RNA concentrations by spectrophotometer. We detected the lowest RNA concentration of 9.2 ± 0.3 ng/µL in d1–2 CSF pool followed by increasing RNA concentrations of 11.1 ± 0.3 ng/µL, 12.9 ± 0.3 ng/µL and 14.6 ± 0.5 ng/µL in d3–4, d5–6 and d7–12, respectively (Figure 2B). The increasing RNA concentrations were surprising, since they did not match the level of haemolysis which were clearly visible by the naked eye as different shades of red, and this often compromises the miRNA analysis [30]. We assessed the haemolysis level by performing reverse transcription and applying the cDNAs to a commercially available quality control (QC) PCR plate containing miR-451 and miR-23a-3p as haemolysis dependent and independent miRNAs, respectively [31]. The difference in threshold cycles (ΔCt = Ct(miR-451) − Ct(miR23a-3p)) of 7 or more indicates a high risk of haemolysis [30]. We found the highest ΔCt values of 8.90 ± 0.19 and 9.76 ± 0.17 in d1–2 and d3–4, respectively, while in d5–6 and d7–12 we detected ΔCt values of 7.61 ± 0.04 and 6.87 ± 0.23, respectively (Figure 2C). These results indicate that d1–2 and d3–4 might be significantly more affected by haemolysis than d5–6 and d7–12.

To better characterise plasma-derived content in CSF pools and their potential association with detected miRNAs, we performed a western blot analysis for albumin and some apolipoproteins (Apo). We detected similar profiles for albumin and ApoAI, with the highest protein levels in d1–2 followed by moderate but comparable levels in d3–4 and d5–6 and low levels in d7–12 (Figure 2D). We noticed moderate and comparable levels of ApoE in d1–2 and d3–4, but higher and comparable levels of ApoE in d5–6 and d7–12. These data show that the CSF during the first four days after sTBI was more compromised with the blood content in comparison to the later days. To assess whether the intracellular content from damaged cells is also present in CSF pools, we analysed several peroxiredoxins (Prdx), which are ubiquitous proteins involved in the antioxidative response in various cells, including erythrocytes and leukocytes [32]. Similar to albumin and apolipoprotein profiles, we detected the highest level of Prdx2 and Prdx6 in d1–2 followed by lower but similar levels at d3–4 and d5–6, and a very low level in d7–12. We noticed very low levels of Prdx1 and Prdx5 at d1–2 and no clearly detectable signals for these two peroxiredoxins in any of the other CSF pools.

### 2.3. Targeted miRNAs Are Present in Post-TBI CSF with Quantities Differing up to a Million-Fold

**Figure 3:**
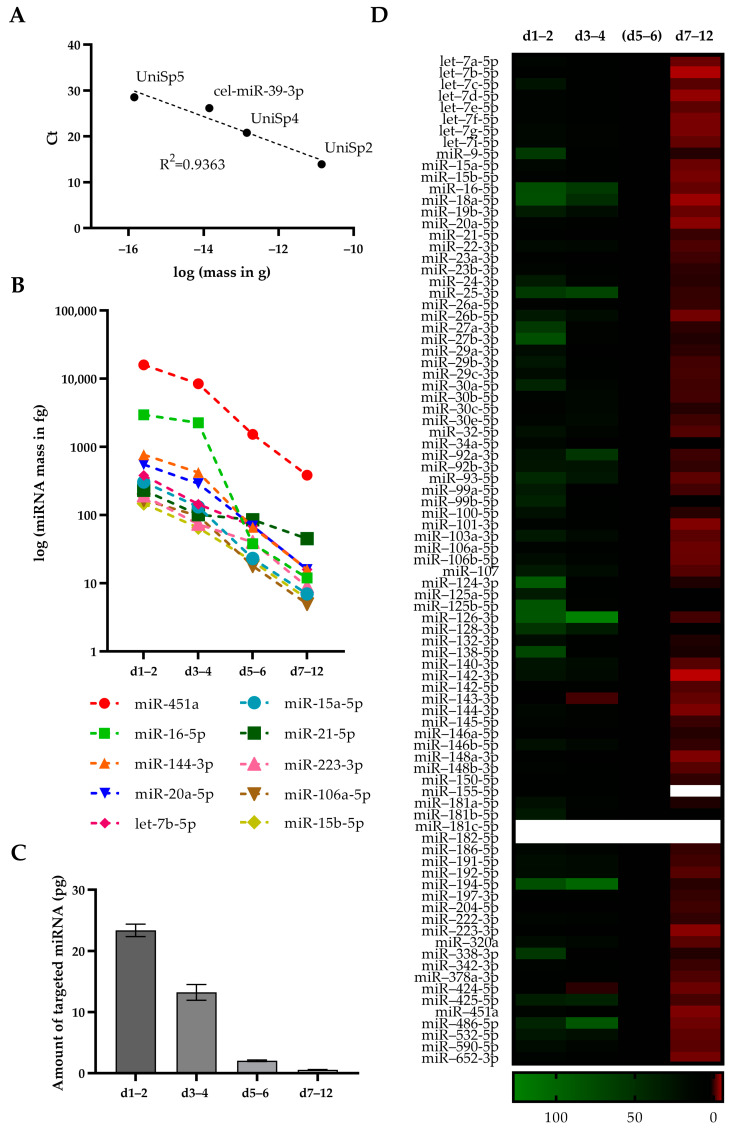
**Targeted microRNAs (miRNAs) are abundant in the early days after severe traumatic brain injury (sTBI).** (**A**) Absolute quantification of targeted miRNAs was performed by standard curves obtained by applying four miRNA spike-ins of pre-defined concentrations. Spike-ins were amplified simultaneously with targeted miRNAs on a PCR-array followed by plotting spike-in amounts against obtained cycle thresholds (Ct). A representative plot is shown. (**B**) Spike-in containing cDNAs from cerebrospinal fluid (CSF) pools of indicated days (d) after sTBI were analysed by PCR-array targeting 87 miRNAs, of which the indicated miRNAs are shown after their quantification based on spike-ins. (**C**) Amounts of targeted miRNAs quantified by RT-PCR were summed for all indicated CSF pools. The mean values of two independent experiments are shown. (**D**) Heat map of targeted miRNAs across indicated CSF pools after absolute quantification followed by normalisation against d5–6 values. Empty boxes denote missing values due to undetected miRNA levels required for normalisation.

After the initial characterisation of total miRNA in CSF pools together with proteins of plasma and cellular origin, we then set out to identify and quantify miRNAs present in CSF after sTBI. To enable the quantification of miRNAs, we utilised spike-in miRNAs (UniSp2, UniSp4 and UniSp5), which were available at a 100-fold concentration difference and were included for detection in the PCR-array to be applied. We added spike-ins in the cDNA synthesis step, thereby preserving their initial concentrations. After performing a real-time PCR we used the resulting Ct values to create standard curves (Figure 3A). We were able to detect all spike-ins in every PCR-array measurement, including the UniSp5 spike-in, which corresponded in the amount of miRNAs present at a very low concentration. We obtained standard curves with R2-values ranging from 0.94 to 0.91 and used them to quantify miRNAs.

We detected and quantified all 87 targeted miRNAs, but their levels differed by more than a million-fold across the four CSF pools (Table S1). Almost all of the 87 targeted miRNAs showed the highest levels at d1–2 except for miR-25-3p, miR-486-5p, and miR-92a-3p, which exhibited the highest levels at d3–4. Based on detected Ct values for d1–2, we could group miRNAs into high (Ct < 25), moderate (Ct 25 to <30), and low (Ct 30 and more) abundant miRNAs, resulting in amounts of approximately 4–16 pg, 2–4 pg and less than 1 pg, and comprising 54, 29 and 4 targeted miRNA sequences, respectively. We could not detect miR-155-5p at d7–12 or miR-181c-5p and miR-182-5p at both d5–6 and d7–12.

We noticed that the following 10 miRNAs are the most abundant across all four CSF pools: miR-451a, miR-16-5p, miR-144-3p, miR-20a-5p, let-7b-5p, miR-15a-5p, miR-21-5p, miR-223-3p, miR-106a-5p and miR-15b-5p (Figure 3B). Although all of these 10 miRNAs decreased in amount with every time point, their charts showed different slopes, with miR-451a and miR-21-5p having the biggest and the smallest slope, respectively. These results might indicate different cellular sources of quantified miRNAs.

The trend of a decreasing amount of miRNA was also visible when we calculated the total mass of the targeted miRNA across all CSF pools (Figure 3C). The highest amount of 23.37 ± 1.03 pg of total targeted miRNA was detected in the d1–2 pool, followed by 13.22 ± 1.29 pg, 2.05 ± 0.12 pg, and 0.55 ± 0.05 pg in d3–4, d5–6 and d7–12, respectively. Taking into account that cDNA synthesis was performed with equal RNA amounts, these results indicate that CSF from later days contains miRNAs not included in the applied PCR-array.

To identify relative changes in individual miRNA levels across all CSF pools, we normalised miRNA levels against d5–6 levels, since the haemolysis at that time point was low in comparison to d1–2 and d3–4 (Figure 3D). The resulting heat map showed a common trend for the majority of miRNAs, with the highest level at d1–2 followed by decreasing levels in later days. These results also indicate that the targeted 87 miRNAs are likely to be of blood origin.

### 2.4. CD81-Enriched Extracellular Vesicles Present in CSF Samples Contain miRNA and Have Increased Size at Days 7–12 after sTBI

**Figure 4:**
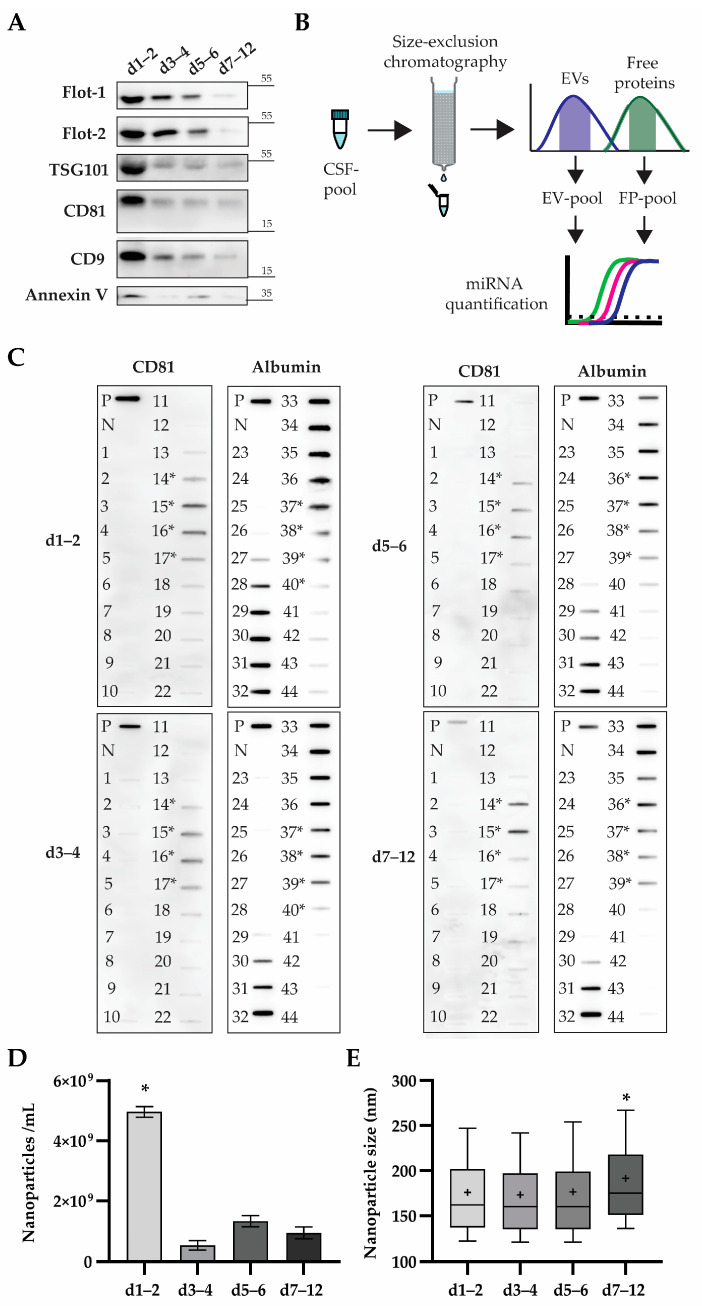
**Cerebrospinal fluids (CSFs) after severe traumatic brain injury (sTBI) contain extracellular vesicles (EVs) which can be clearly separated from free proteins (FPs) by size exclusion chromatography (SEC).** (**A**) Listed CSF pools were analysed by western blot and the immunodetection of depicted proteins with indicated sizes. Representative immunoblots are shown. (**B**) Scheme of major steps in analysing microRNAs (miRNAs) from EVs and FPs obtained by SEC. (**C**) CSF pools were separated by SEC, resulting in fractions applied to nitrocellulose membranes by slot-blot and analysed by the immunodetection of CD81 and albumin. The corresponding CSF pool was lysed and applied as a positive control (PC), while phosphate buffered saline (used as SEC mobile phase) was applied as a negative control (NC). Numbers indicate individual SEC-fractions, and asterisks mark the SEC-fractions pooled for further analyses. (**D**) Nanoparticle concentration was determined in the pool of CD81+ SEC-fractions by tunable resistive pulse sensing (TRPS). Mean values with standard deviations from three measurements performed at two pressure conditions are shown. * *p* < 0.001 for d1–2 in comparison to all later CSF pools, one-way ANOVA with Tukey’s test for multiple comparisons. (**E**) Nanoparticle diameter was determined in a pool of CD81+ positive SEC-fractions by TRPS. Each sample was measured three times at two pressure conditions. Values are shown as box plots marking the median (bar), 25th and 75th percentiles (box), the 10th and 90th percentiles (whiskers), and mean value indicated as “+”. * *p* < 0.001, non-parametric ANOVA (Kruskal-Wallis test) with Dunn’s test for multiple comparisons.

To test whether some of the 87 targeted miRNAs were EV-cargo in CSF after sTBI, we first characterised the EV populations present in CSF pools (Figure 4A). By applying western blot analyses, we could detect protein markers for exosomes, i.e., medium size EVs, as per recently recommended nomenclature [33]. CD9, CD81, Flotillin-1 and -2 and TSG101 were present at high levels at d1–2, followed by a moderate decrease in levels for CD9, Flotillin-1 and -2, and a sharp decrease for CD81 and TSG101 at d3–4 and d5–6. All five EV-protein markers were detected at very low levels at d7–12. We could also detect Annexin V, the protein marker of apoptotic bodies, albeit at low levels at d1–2 and d5–6, and hardly detectable at levels at 3–4 and d7–12. These data suggest that apoptotic bodies are only a minor population of EVs in analysed CSF- pools, while a substantial portion of CD9, Flotillin-1 and -2 positive EVs might have a plasma origin, since these proteins were detected at higher levels in haemolysis compromised CSF pools. Interestingly, CD81 and Tsg101 showed low but constant and comparable levels at d3–4, d5–6 and d7–12, suggesting that haemolysis might not be the major source of CD81 and Tsg101 positive EVs in those CSF pools.

After characterising EV populations, our next goal was to separate the CSF pools into EV- and free protein (FP)-enriched fractions and use them for miRNA isolation and quantification (Figure 4B). For CSF pool separation, we applied size-exclusion chromatography (SEC) followed by immunodetection of CD81 and albumin in SEC-fractions as previously shown to be an effective way of EV isolation and detection [34]. In a total of 44 fractions collected in each SEC, we detected CD81 and albumin in fractions 14–17 and 28 and/or later, respectively (Figure 4C). These data demonstrate that CD81 and albumin enriched fractions were clearly separated in all four CSF pools.

After pooling CD81+ fractions, we measured the numbers and sizes of isolated EVs by tunable resistive pulse sensing (Figure 4D,E). We detected in d1–2 (4.96 ± 0.18) × 10^9^ particles/mL, which was significantly higher in comparison to later days where we found concentrations of (0.52 ± 0.15) × 10^9^, (1.32 ± 0.19) × 10^9^ and (0.93 ± 0.19) × 10^9^ nanoparticles/mL in d3–4, d5–6 and d7–12, respectively. Interestingly, EV sizes were comparable in d1–2, d3–4, and d5–6, with a median diameter of 162 nm (IQR 137–202 nm), 160 nm (IQR 137–202 nm) and 160 nm (IQR 135–197 nm), respectively, but EVs at d7–12 measured 175 nm (IQR 151–218 nm) in diameter, which was significantly higher in comparison to EVs from earlier pools (Figure 4D and Figure S1).

### 2.5. Only a Smaller Portion of Targeted miRNAs Are Potential Cargo of Extracellular Vesicles

**Figure 5:**
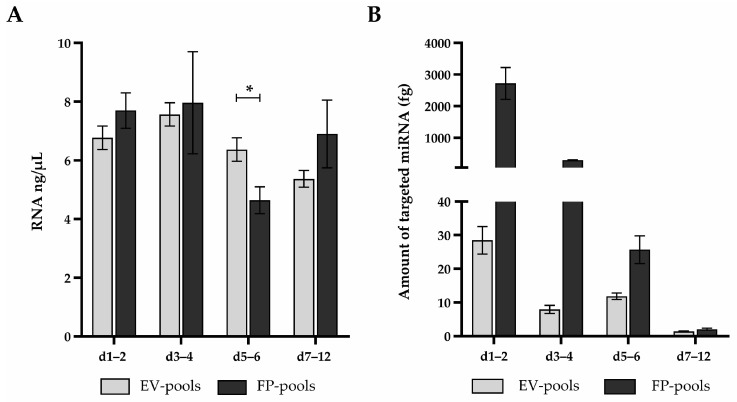
**Targeted microRNAs (miRNAs) are present in extracellular vesicles (EVs) and free proteins (FPs) separated from cerebrospinal fluid (CSF), but only as a small portion of detected RNA.** (**A**) miRNA was isolated from indicated CSF pools after they were separated by size exclusion chromatography (SEC) to EVs and FPs. RNA concentration was determined by spectrophotometric measurement. Mean values and standard deviations from two measurements are shown. * *p* = 0.008 by unpaired *t*-test. (**B**) Targeted miRNAs were quantified in EV- and FP-pools obtained after SEC, followed by a PCR-array and absolute quantification. Detected miRNA amounts were summed for each indicated time point. Mean values and standard deviations from two measurements are shown.

To discover if targeted miRNAs are cargo of post-TBI EVs in CSF, we isolated miRNA from EV- and FP-enriched fractions obtained by SEC and detected RNA with mean values in a range between 4.6 and 8.0 ng/µL in total, including for both isolate types and across all of the originating CSF pools (Figure 5A). After performing cDNA synthesis and real-time PCR, we quantified targeted miRNAs in EV- and albumin-enriched fractions (Table S2).

Similar to the miRNA quantification in CSF pools, we detected the highest amounts of targeted miRNAs in d1–2 followed by decreasing amounts in the following time points (Figure 5B). However, detected miRNA levels were roughly 1000-fold lower in comparison to CSF pools ranging from approximately 2.2 pg to less than 0.01 fg, and were mostly lower in the EV-pool in comparison to the FP-pool for the corresponding time point. The highest levels were detected in FP-pools of d1–2 for miR-451a, miR-144-3p, and miR-20a-5p in amounts of 2.22 pg, 0.16 pg and 0.01 pg, respectively. We could quantify levels in all four time points for only 20 miRNAs in FP-pools, while the majority of d5–6 and d7–12 levels were below detectable values.

To assess the enrichment of quantified miRNAs in FP- and EV-pools, we determined corresponding ratios for available quantities and only considered ratios with at least a 5-fold change (Table 2). In line with the highest miRNA levels detected in FP-pools at d1–2 and 3–4, we found that 59 and 35 of the targeted miRNAs are enriched up to approximately >200-fold and >300-fold, respectively. We found only 3 miRNAs to be enriched in the FP-pool at d5–6 and d7–12 up to 12-fold and 14-fold, respectively. In contrast to FP-enriched miRNAs, we detected much fewer miRNAs to be EV-enriched. We found miR-223-3p to be exclusively EV-enriched at d5–6 (26-fold) and d7–12 (16-fold), followed by miR-30b-5p (9-fold) and miR-92b-3p (5-fold) being solely enriched at d1–2. We also detected miR-204-5p to be enriched only in EV-pools at d3–4 (55-fold) and d5–6 (42-fold). Additionally, d5–6 contained miR-142-3p (54-fold), miR-125b-5p (9-fold) and miR-22-3p (8-fold) as being uniquely enriched in the EV-pool. However, other miRNAs found to be EV-enriched at d3–4 (miR-99-a-5p) and at 5–6 (12 miRNAs underlined in Table 2) were also found to be FP-enriched at some other time points. This might be due to technical reasons, such as the incomplete separation during SEC or due to the different biology of some miRNAs, whereby the same miRNA originating from different sources might be associated with either EVs or other carriers.

## 3. Discussion

Severe traumatic brain injury (sTBI) offers a unique opportunity to investigate complex pathophysiological processes unfolding in the brain early after the injury. One of the measures in sTBI treatment includes the placement of an external ventricular drain (EVD), which provides access to the intracranial cerebrospinal fluid (CSF). Moreover, the intracranial CSF obtained by EVD might also contain signals required for the recovery and/or normal functioning of the brain. We hypothesized that microRNAs (miRNAs) released either passively from damaged tissue or actively from living cells could be such signals. Our results describe CSF dynamics in the context of miRNA types and quantities during the early days after the injury.

The study included five sTBI patients with a different number of consecutive CSF samples collected after the injury (Figure 1). The reason for missing CSF samples was the removal of the EVD at the indication of the neurosurgeon due to clinical improvement and, in one case, due to the patient’s fatal outcome. Patient outcome was monitored through the international Glasgow Outcome Scale (GOS), showing overall values of four and five points for recovered patients after the sTBI (Table 1). These scores indicate moderate and mild neurological disability with minimal to no neurological deficits present. Therefore, the CSF samples included in the study originate from the early phase of neurological recovery. However, the number of included sTBI patients and corresponding CSF samples is low, which presents the main limitation of the study.

We combined consecutive CSFs to enable PCR-array based screens—including CSF before and after separation by size-exclusion chromatography (SEC)—from different time points after sTBI. The combining of the CSFs samples into pools resulted in the dilution of the originating samples. While the d1–2, d3–4, and d5–6 CSF pools provide average miRNA amounts for the two days (d) included, the d7–12 CSF pool provides average miRNA amounts for the six days included. Therefore, it is very likely that at least some miRNAs at different time points were detected at low levels or not at all due to their dilution in CSF pools, posing an additional limitation of the study.

CSF after sTBI has a specific composition due to the cell damage and impaired blood-brain barrier as part of the TBI pathophysiology. This also reflects the miRNA content in the CSF after sTBI, with many miRNAs most likely originating from the blood, as our and other groups have shown [30]. We found the highest miRNA levels in early days after sTBI represented by d1–2 and d3–4 CSF pools. Both CSF pools have also displayed high levels of haemolysis, as detected by real-time PCR. Our data suggest that the targeted miRNAs, although chosen as commonly present in CSF and presumably of central nervous system (CNS)-origin, might be mainly originating from damaged blood cells, predominantly erythrocytes. This is supported by miR-451a, whose d1–2 and d3–4 levels were approximately 20 ng and 10 ng, respectively, and they clearly stood out in comparison to other miRNAs (Table S1). miR-451a is highly expressed in erythrocytes and is enriched in erythrocyte EVs, which can also pass through the impaired blood-brain barrier [35,36]. miR-451a can promote apoptosis in neurons, thereby contributing to sTBI pathophysiology, but it can also mediate neurite outgrowth and regulate the endothelium, as shown in animal studies [37,38,39]. Other highly abundant miRNAs at d1–2 and d3–4 included miR-16-5p, miR-144-3p, miR-20a-5p, let-7b-5p, miR-15a-5p, miR-21-5p, miR19b-3p, miR-223-3p, miR-106a-5p, and miR-15b-5p, all of which were reported to be expressed at high levels mainly in veins and arteries, confirming earlier findings where most of the mentioned miRNAs have been detected from serum and plasma samples after brain tissue and blood vessel injury [9,35]. Some of the listed miRNAs might further aggravate sTBI by different mechanisms. Upregulated miR-16p-5p and miR-15a-5p can target BCL-2 and induce apoptosis in neuronal cells, and can also negatively impact the endothelium in the blood-brain barrier [40,41,42]. In vivo and in vitro studies demonstrated that overexpressed miR 144-3p can impair neuron viability and cognitive functions in a TBI model as well as result in a reduced response to oxidative stress [43,44]. In the ischemic and hypoxia models, miR-20a-5p was shown to downregulate NeuroD1 and Kif5A, which are required for the growth of dendrites, and the elimination of neurotoxic substances, respectively [45,46]. However, other in vitro studies show that miR-20a-5p can have a neuroprotective effect and stimulate axonal growth [47,48,49]. The positive affect in sTBI might be also attributed to miR-21-5p, which was demonstrated in animal models to inhibit apoptosis and promote angiogenesis, resulting in better neurological outcomes after TBI [45]. Additional neuro-protection is associated with miR-106a-5p, which can participate in the amelioration of oxidative stress and brain injury after intracerebral haemorrhage [50]. MiR-223-3p was shown to downregulate the NLRP3 inflammasome, which could lead to reduced brain oedema and improved neurological function [51]. Moreover, miR-233-3p is able to inhibit apoptosis in brain microvascular endothelial cells (BMCEs), and thereby provides a protective role to the integrity of the blood brain barrier, and the maintenance of the CNS microenvironment balance [52]. MiR-let-7b-5p was singled out as an miRNA with high expression in the brain tissue, arachnoid sheath, and spinal cord [53]. The effects of let-7b-5p can also be detrimental in TBI with CSF-contained let-7b-5p, which is shown to induce neurodegeneration mediated by neural Toll-like receptor (TLR) 7 [54]. Furthermore, let-7a/b-5p containing exosomes from microglia can induce apoptosis in neurons [55]. In addition, the mentioned miRNAs are detectable in different expressions in other tissues, meaning that they could possibly be products of affected organs such as lungs, kidneys, pancreas, spleen, and testicles that are afflicted with some other disease [29,53,56]. Moreover, we cannot exclude that some underlying diseases were present in sTBI patients, which might affect miRNAs, but have not been known at the time of sampling.

Although all miRNAs showed a general trend of decreasing amounts towards later CSF pools with the consistent decrease of haemolysis, we noted that highly abundant miRNA levels tend to decline at different rates (Figure 3B). This would indicate that the detected miRNAs might originate from different cells. Although a more detailed characterisation of cellular markers was out of scope, all CSF pools contained peroxiredoxins 2 and 6 (Figure 2D). Both peroxiredoxins are ubiquitously expressed in nucleated cells, including leucocytes [57]. Notably, inflammation is a common complication in sTBI patients as a result of signalling pathways in secondary brain injury, and also as a general reaction of the human organism to a stressful situation [4,58,59]. A further source of miRNA in CSF after sTBI might be blood-derived extracellular miRNAs due to an impaired blood-brain barrier [52]. We could detect albumin and apolipoproteins across all analysed CSFs (Figure 2D). Both albumin and apolipoproteins were described as carriers of extracellular miRNA [60,61].

Our miRNA quantification suggests that other miRNAs, not necessarily of blood-origin, could be present in CSF. We found that the total amount of targeted miRNAs has made up only a small portion of miRNAs applied in cDNA synthesis. This particularly applies to the d5–6 and d7–12 CSF pools in which haemolysis was not pronounced so much as in earlier CSF pools (Figure 2C). To see if we can improve the detection of targeted miRNAs, we separated the CSF pools into extracellular vesicles (EVs) and free proteins (FP) to obtain more homogeneous sources of miRNAs. Both EVs and FPs were shown to be carriers of miRNAs, but EVs are especially promising for two reasons. First, miRNAs contained in EVs have higher stability. Second, EVs provide a specific delivery of their cargo, since they contain proteins and lipids on their surface that are able to interact with the targeted cell [23].

Before isolating EVs, we analysed common EV-protein markers in CSF and found high levels of Flotilin-1 and -2, TSG101, CD81 and CD9 at d1–2, followed by decreasing amounts in later days (Figure 4A). This is consistent with the blood content and haemolysis levels described in previous results, since all of these proteins are ubiquitously expressed in cells, including blood cells. We detected low levels of annexin V at d1–2 and d5–6, indicating that these CSF pools were affected by apoptosis, which is also a well-recognised part of TBI pathophysiology. Apoptotic bodies are a type of EVs of 50 to 5000 nm in diameter [62]. Due to the partial overlapping with medium sized EVs of approximately 150 to 200 nm in diameter analysed in this study (Figure 4E), we cannot exclude the possibility that a portion of the apoptotic bodies were co-isolated and therefore contributed to the detected miRNAs. We isolated EVs by size-exclusion chromatography, a method suitable for the isolation of total EVs and that was also shown to result in intact EVs [63]. Furthermore, we have previously shown that the Sepharose CL-6B applied here is effective in isolating EVs from CSF [34]. This has proven to be crucial for this study, since we were able to isolate RNAs in concentrations sufficient for cDNA analysis from both FPs and EVs (Figure 5A).

We did not detect all targeted miRNAs after SEC, and we noticed much lower amounts in comparison to CSF pools (Table S2). This is expected, since the analyses only included chosen SEC-fractions based on the detection of selected proteins, with CD81 and albumin used as markers for EV- and FP-fractions, respectively (Figure 4A). A majority of miRNAs were detected in FP-fractions, with miR-451a as the most abundant miRNA, similar to the CSF pool analyses (Table 2). Considering the previously described issues with haemolysis and damage to other tissues after sTBI, it is tempting to speculate whether free protein associated miRNAs are released from lysed cells and not as a result of secretion from intact cells. We found some miRNAs to be both EV- and FP-abundant depending on the analysed days. This miRNA ambiguity might be due to a technical reason such as incomplete separation of EVs and FPs. Another possibility is that the same miRNA can be present in both EV- and FP-form depending on the cell source. Interestingly, several miRNAs, including miR-142-3p, miR-204-5p, and miR-223-3p, showed unique and strong enrichment in EVs at d7–12 when haemolysis was less present (Table 2).

Taken together, we here described the quantitative changes of miRNAs in CSF of patients recovering from sTBI. We showed the high impact of haemolysis on 87 targeted miRNAs in the first four days. However, the CSF from later days contained enlarged EVs carrying RNA, which is yet to be characterised. Our results emphasise the dynamic nature of sTBI and the need to further analyse CSF at later days when neuro-recovery signals, such as miRNAs, might be present.

## 4. Materials and Methods

### 4.1. Patients

Research included five patients with severe traumatic brain injury (sTBI) treated at the Anaesthesiology, Intensive Medicine and Pain Treatment Clinic at the Clinical Hospital Centre Rijeka and at the Clinic for Anaesthesia, Reanimation, Intensive Care Medicine and Pain Treatment at Pula General Hospital, Croatia. Clinical requirements for sTBI were Glasgow Coma Scale (GCS) ≤ 8, neurological physical examination, and neuroradiological imaging of the brain (Multi Slice Computer Tomography, MSCT). The study was approved by the Institutional Review Board of both hospitals, and informed consent was signed by the family member or a legal representative due to the patient being unconsciousness and under critical care management. Treatment included placement of an external ventricular drain (EVD) at the indication of an attending neurosurgeon for the purpose of intracranial pressure (ICP) monitoring and management. The exclusion criteria were patient age under 18 years, since it was a study conducted on adults, and patient age over 80 years, due to inevitable physiological changes in the brain. Patients with susceptible immune, malignant and infectious conditions due to potential interaction at the level of pathophysiological signalling pathways were also excluded from the study. No specific clinical parameters were considered or monitored for the purposes of this study. The control group was not included for two reasons. Firstly, applying EVD in healthy subjects, which would provide cerebrospinal fluid (CSF) from the same anatomic position as in sTBI patients, is not possible due to ethical reasons. Secondly, CSF obtained by lumbar punction includes a risk of different CSF composition due to the changed anatomical location. Additionally, lumbar-CSF has a further risk of potential contamination with other cellular content in comparison with EVD-derived CSF. Patient treatment outcomes were monitored after three months using the international Glasgow Outcome Score (GOS).

### 4.2. CSF Collection and Sample Pooling

CSF was sampled from the EVD and its drainage chamber daily, starting from 24 h after its placement. Samples were collected during twelve days after the sTBI in low-protein binding tubes (Eppendorf, Hamburg, Germany). The indication for stopping the sampling was the removal of the EVD indicated by the attending neurosurgeon or the fatal outcome of the patient. After sampling, CSF was stored at −80 °C. Samples were pooled in equal volumes to obtain four consecutive CSF pools (Figure 1).

### 4.3. Western Blot

Generated CSF pools were mixed with 5× Laemmli buffer (1M Tris HCl pH 6.8, 50% glycerol (*v*/*v*), 10% SDS (*w*/*v*), 0.05% bromophenol blue (*w*/*v*), 2-mercaptoethanol) and boiled at 95 °C for 10 min. Pooled CSF together with a protein standard (PageRuler™ Prestained Protein Ladder, Thermo Scientific, Waltham, MA, USA) were loaded onto 12% polyacrylamide gel and electrophoresed (MiniVE SE 300, Hoefer, Holliston, MA, USA) in 1x running buffer (25 mM Tris, 192 mM glycine and 0.1% SDS, pH 8.3) on 90 V to 150 V. Separated proteins were transferred to 0.2 µm nitrocellulose membrane (Global Life Sciences Solutions Operations UK Ltd., Little Chalfont, UK) at a constant voltage of 70 V for an hour and a half in a 1x transfer buffer (25 mM Tris, 192 mM glycine and 20% methanol). Following the protein transfer, membranes were stained with Ponceau S (0.1% Ponceau S in 5% acetic acid), blocked with 5% milk in Tris-buffer saline (TBS, 20 mM Tris and 150 mM NaCl) for 15 min, and incubated with rabbit monoclonal antibodies against albumin (#4929), apolipoprotein E (#13366), CD9 (#13403), CD81 (#52892), Flotilin-1 (#18634), Flotilin-2 (#3436) and TSG101 (#72312) and mouse monoclonal antibody against apolipoprotein AI (#3350), and diluted 1:1000 in 5% bovine serum albumin (Cell Signalling Technology, Danvers, MA, USA)/TBS-T (TBS supplemented with 0.1% Tween 20) over-night at 4 °C. Membranes were washed three times for 5 min in TBS-T and incubated with horseradish peroxidase-linked anti-rabbit (#7074) or anti-mouse (#7076) secondary antibodies at room temperature for 30 min (all listed primary and secondary antibodies were obtained from Cell Signalling Technology, Danvers, MA, USA). After additional washing in TBS-T, the signal was visualised using SignalFire Plus ECL Reagent or SignalFire Elite ECL Reagent (Cell Signalling Technology, Danvers, MA, USA) and imaged with an ImageQuant LAS 500 CCD imager (GE Healthcare Bio-Sciences AB, Uppsala, Sweden).

### 4.4. Size-Exclusion Chromatography

Size-exclusion chromatography (SEC) was performed on CSF pools as previously described [34]. Briefly, samples were separated using Sepharose-CL-6B (GE Healthcare Bio-Scences AB, Uppsala, Sweden), and packed in a 1.5 × 50 cm glass column equipped with a flow adaptor (Bio-Rad Laboratories, Hercules, CA, USA). Prior to separation, the column was equilibrated with sterile PBS (Life Technologies Corporation, Grand Island, NY, USA), which was used as a running buffer. A total volume of 4 mL of each CSF pool was applied onto a gravity-flown column by 5 mL sterile plastic syringe, and 46 fractions of 1.5 mL were collected in low-protein-binding tubes (Eppendorf, Hamburg, Germany). The SEC column was flushed with at least two bed volumes of PBS between each SEC run.

### 4.5. Slot blot

Collected SEC fractions were mixed with a 5x Laemmli buffer without glycerol and boiled at 95 °C for 10 min. Nitrocellulose membrane (Global Life Sciences Solutions Operations UK Ltd., Little Chalfont, UK) was soaked in distilled water and placed in slot blot apparatus (Hoefer Inc., Richmond, CA, USA), connected to a vacuum pump. SEC fractions in volume of 300 µL were applied to the slot blot, pulled through a membrane by a vacuum pump, and rinsed three times with 1 mL of PBS (Life Technologies Corporation, Grand Island, NY, USA). The membrane was then removed from the slot blot apparatus, stained with Ponceau S, blocked in 5% milk, and blotted for CD81, as described above. The signal was visualised using SignalFire Elite ECL Reagent (Cell Signalling Technology, Danvers, MA, USA) on an ImageQuant LAS 500 (GE Healthcare Bio-Sciences AB, Uppsala, Sweden).

### 4.6. Tunable Resistive Pulse Sensing Analysis

Tunable resistive pulse sensing (TRPS) analysis of the size distribution and concentration of nanoparticles in the CD81+ pool of SEC fractions was conducted on a qNano Gold platform (Izon Science, Christchurch, New Zealand). The nanopore was wetted, coated and equilibrated with an Izon reagent kit as per the manufacturer’s instructions. The calibration was performed with 200nm carboxylated polystyrene beads (CPC200, Izon Science, Christchurch, New Zealand), diluted 500-fold in filtered electrolyte solution, and stretch was adjusted until the blockade magnitude for calibration particles averaged between 0.25 and 0.3 nA. Measurements were conducted at a constant 46.00 mm stretch and a baseline current at approximately 125 nA. Samples were diluted twofold in filtered electrolyte solution and vortexed for 30 s prior to TRPS measurements. Each sample was measured at two pressure steps of 5 and 2.5 mbar, in triplicate. At least 500 particles were recorded for each measurement. Before each measurement, the nanopore was washed with the electrolyte solution at a pressure of 20 mbar to avoid cross-contamination between samples. Data processing and analysis were performed in Izon Control Suite v3.4 software (Izon Science, Christchurch, New Zealand). A concentration fraction from 110 to 420 nm was applied for all measurements prior to any data analysis.

### 4.7. RNA Isolation

Isolation of RNA from total CSF-, EV-, FP- pools was performed using Norgen’s Urine microRNA Purification Kit (Cat. No./ID:29000, Norgen Biotek Corp., Thorold, ON, Canada), as it was previously shown to be efficient in miRNA isolation from CSF [64]. According to the manufacturer’s recommendation, 1 mL of the CSF sample was used for the isolation procedure, while keeping the samples on ice. The protocol started with the lysis procedure adding the Lysis Buffer A, provided by the manufacturer, to the CSF aliquot and vortexing for 15 s, followed by the addition of 96–100% ethanol provided by the user to the lysate and vortexing for 10 s. Binding on the columns was then performed by centrifugation (Eppendorf, Hamburg, Germany) on 4 °C and 8000 RPM for 1 min until the entire lysate has been loaded onto the column. Columns were assembled with the provided collection tubes. The column was then washed applying the provided Wash Solution A prior to adding of 42 mL of 96–100% ethanol to the solution. Washing was performed by centrifugation for 1 min at 14.000 RPM in three repeated steps. The drying of the column was achieved by centrifugation at 2 min and 14.000 RPM. Columns were then assembled with the manufacturer’s 1.7 mL elution tubes and 30 µL of provided Elution Solution A was added to the columns and incubated at room temperature for 20 min. The following final step was centrifugation for 2 min at 2000 RPM and an additional 2 min at 14.000 RPM. The obtained amount of sample (about 28 µL) was stored in low-protein binding tubes (Eppendorf, Hamburg, Germany). RNA quantification was performed by the application of 1 µL of sample onto the spectrophotometer (NanoPhotometer^®^ P330, Implen GmbH, München, Germany) in triplicates for every sample.

### 4.8. cDNA Synthesis and Real-Time PCR

An amount of 20 ng of miRNA isolated from each CSF-, EV- and albumin pool was reverse transcribed in 20 µL reactions using miRCURY Locked Nucleic Acid (LNA) RT Kit (Cat. No./ID: 339340, Qiagen Sciences, Germantown, MD, USA) with a thermal cycler (Applied Biosystems, Singapore, Singapore). A known amount of 5 different RNA spike-ins (UniSp2, UniSp4, UniSp5 and cel-miR-39-3p provided in Qiagen RNA Spike-In Kit, For RT, Cat. No./ID: 339390 and UniSp6 provided with miRCURY LNA RT Kit) was added to each reaction prior to the cDNA synthesis step for quality control and quantification purposes. RNA quality and reverse transcription were assessed by PCR-based QC array (miRCURY microRNA QC PCR Panel, Cat. No./ID: 339345, Qiagen Sciences, Germantown, MD, USA) before proceeding with miRNA analysis. miRNA profiling was undertaken using a PCR-based array on a 96-well plate (miRCURY LNA Human CSF Exosome Focus miRNA PCR Panel, Cat. No./ID 339325PF-1/YAHS-124Y, Qiagen Sciences, Germantown, MD, USA) and a real-time PCR machine (7300 real time PCR system, Applied Biosystems, Singapore, Singapore).

## 5. Conclusions

This study describes quantities of 87 miRNAs in the cerebrospinal fluid (CSF) of patients with severe traumatic brain injury (sTBI). Absolute quantification was performed at four time points over twelve days after the injury, providing an insight into the dynamics of miRNAs of putative CSF origin.

We showed that CSF contains miRNAs throughout the twelve days after sTBI. The targeted 87 miRNAs were detected at very different levels in CSF, with their combined amounts making only a small portion of total miRNA present in the CSF. Furthermore, our results indicated that the targeted miRNAs most likely originate from lysed erythrocytes and potentially other damaged cells. We also demonstrated that the majority of targeted miRNAs were associated with free proteins, while only some miRNAs were enriched in extracellular vesicles (EVs). However, EVs were found in all analysed CSF pools, and their content included miRNAs.

Several major limitations of the study should be emphasized. First of all, the number of subjects was low and the analysed cohort of patients was heterogenous with regard to the underlying intracranial pathology, including the type of haemorrhage, site of intraparenchymal damage, and whether diffuse axonal injury was detected. This is especially critical since blood-derived content in CSF after sTBI largely influenced the amount and type of miRNAs detected in the study. Furthermore, no clinical parameters were included in the study, and therefore their potential correlation with the study findings is missing. Taken together, this is a preliminary miRNA study which further corroborates the complex dynamics of sTBI in the acute phase.

Future studies should identify other miRNAs present in CSF after sTBI, including the CSF-EVs. Moreover, in contrast to the current study based on pooled CSF samples d1–2, d3–4, d5–6 and d7–12, novel research should involve more frequent time points and the investigation of unpooled samples. This might enable the detection of novel miRNAs in CSF and could improve the understanding of intracranial processes after sTBI.

## Figures and Tables

**Table 1:** Demographics, injury and recovery of severe traumatic brain injury (sTBI) patients included in the study.

Patient	Age	Gender	Mechanism of Injury	Intracranial Pathology	GCS ^1^	GOS ^2^
1	44	M	fall from height	epidural haematoma	5	4
2	49	F	motor vehicle accident	intracerebral haematoma, subdural haematoma	5	4
3	33	M	pedestrian in car accident	subdural haematoma, focal brain injury (frontal, temporal, occipital)	3	4
4	24	M	motor vehicle accident	diffuse axonal injury	5	5
5	19	M	fall from height	subdural haematoma, subarachnoid haemorrhage	3	1

^1^ Glasgow Coma Scale score at admission. ^2^ Glasgow Outcome Scale score three months after discharge.

**Table 2:** Enrichment of targeted microRNAs (miRNAs) in free protein (FP)- and extracellular vesicle (EV)-pools from cerebrospinal fluid (CSF) of patients with severe traumatic brain injury. miRNAs enriched in both EV- and -FP-pools are underlined.

CSF	EV-Enriched (Fold)		FP-Enriched (Fold)							
d1–2	miR-30b-5p miR-92b-3p	(9) (5)	miR-20a-5p miR-29b-3p miR-424-5p miR-451a miR-106a-5p miR-148a-3p let-7i-5p let-7b-5p miR-93-5p miR-144-3p miR-106b-5p miR-101-3p miR-148b-3p miR-29c-3p miR-652-3p	(269) (253) (196) (179) (166) (159) (139) (110) (96) (82) (81) (80) (71) (67) (65)	miR-18a-5p miR-29a-3p miR-15a-5p miR-194-5p miR-192-5p miR-100-5p miR-132-3p miR-26b-5p miR-32-5p miR-107 let-7g-5p miR-15b-5p miR-23a-3p miR-143-3p miR-103a-3p	(64) (53) (40) (40) (36) (33) (32) (31) (29) (29) (28) (28) (27) (23) (22)	let-7d-5p miR-142-5p miR-21-5p miR-532-5p let-7e-5p miR-425-5p mir-9-5p miR-25-3p miR-99b-5p miR-320a miR-124-3p miR-27b-3p let-7f-5p miR-338-3p miR-146b-5p	(22) (22) (22) (22) (20) (18) (18) (16) (16) (14) (14) (14) (13) (13) (13)	miR-34a-5p miR-16-5p miR-19b-3p miR-99a-5p miR-590-5p miR-27a-3p let-7c-5p let-7a-5p miR-23b-3p miR-222-3p miR-146a-5p miR-140-3p miR-126-3p miR-486-5p	(13) (12) (10) (10) (10) (9) (9) (9) (9) (9) (7) (6) (6) (5)
d3–4	miR-204-5p miR-22-3p miR-99a-5p	(55) (6) (5)	let-7b-5p miR-20a-5p miR-106a-5p miR-451a let-7g-5p miR-101-3p miR-148a-3p miR-93-5p miR-26b-5p	(336) (181) (123) (56) (51) (49) (44) (39) (39)	miR-107 let-7f-5p miR-106b-5p miR-103a-3p let-7a-5p let-7i-5p miR-148b-3p miR-486-5p let-7d-5p	(36) (35) (28) (27) (25) (22) (20) (19) (18)	miR-15b-5p miR-425-5p miR-142-5p miR-23a-3p miR-16-5p miR-18a-5p miR-25-3p miR-32-5p miR-92a-3p	(18) (16) (15) (15) (15) (14) (14) (12) (12)	miR-320a miR-144-3p miR-191-5p miR-26a-5p miR-15a-5p let-7c-5p miR-192-5p miR-140-3p	(11) (10) (9) (9) (7) (7) (7) (5)
d5–6	miR-142-3p miR-204-5p miR-223-3p miR-92a-3p miR-338-3p miR-103a-3p miR-486-5p miR-125b-5p miR-25-3p miR-124-3p miR-22-3p miR-16-5p let-7c-5p miR-27a-3p miR-19b-3p miR-27b-3p miR-191-5p	(54) (42) (26) (22) (18) (11) (11) (9) (9) (9) (8) (7) (7) (6) (7) (6) (6)	miR-20a-5p let-7b-5p miR-106a-5p	(12) (11) (6)						
d7–12	miR-223-3p	(16)	let-7b-5p miR-20a-5p miR-106a-5p	(14) (11) (8)						

## Data Availability

Not applicable.

## Supplementary Materials

The following supporting information can be downloaded at: https://www.mdpi.com/article/10.3390/ijms24054751/s1.
