# Supplementary material for: Real-Time PCR Quantification of 87 miRNAs from Cerebrospinal Fluid: miRNA Dynamics and Association with Extracellular Vesicles after Severe Traumatic Brain Injury
Source: Int J Mol Sci. 2023 Mar 1;24(5):4751. doi: 10.3390/ijms24054751 (PMC10003046; doi:10.3390/ijms24054751)
Supplement: Supplementary file 1 [file ijms-24-04751-s001.zip › Figure S1.pdf]

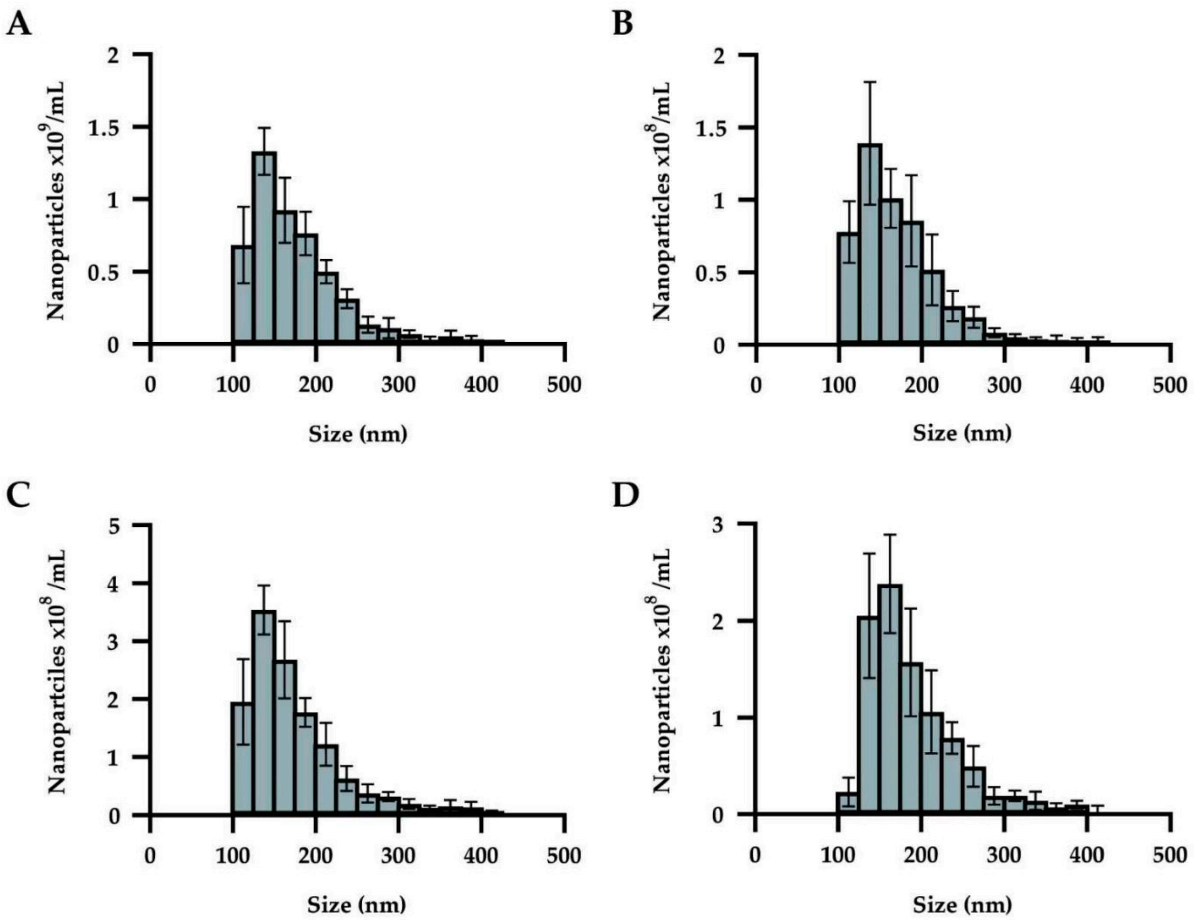

Figure S1. Size and concentration of nanoparticles isolated by size exclusion chromatography from cerebrospinal fluid (CSF) of patients with severe traumatic brain injury (sTBI). Nanoparticles from days (d) d1−2 (A), d3−4 (B), d5−6 (C) and d7−12 (D) after sTBI were quantified by tunable resistive pulse sensing. Shown are mean values and standard deviations of three measurements.
