# Supplementary material for: Real-Time PCR Quantification of 87 miRNAs from Cerebrospinal Fluid: miRNA Dynamics and Association with Extracellular Vesicles after Severe Traumatic Brain Injury
Source: Int J Mol Sci. 2023 Mar 1;24(5):4751. doi: 10.3390/ijms24054751 (PMC10003046; doi:10.3390/ijms24054751)
Supplement: Supplementary file 1 [file ijms-24-04751-s001.zip › Table S1.pdf]

Table S1. Amounts of targeted microRNAs (miRNAs) in cerebrospinal fluid pools obtained at indicated days (d) from patients with severe traumatic brain injury.

Abundance	miRNA mass (d1–2, d3–4, d5–6, d7–12) in fg					
	miR-451a	(16010, 8426, 1524, 385)	miR-125b-5p	(61, 5.6, 0.7, 1.2)	miR-148a-3p	(15, 8.0, 6.0, 1.5)
	miR-16-5p	(2962, 2262, 38, 12)	miR-338-3p	(59, 1.9, 1.1, 0.7)	miR-99a-5p	(15, 0.9, 0.5, 0.2)
	miR-144-3p	(764, 422, 66, 16)	let-7g-5p	(49, 29, 3.8, 1.0)	miR-100-5p	(14, 1.4, 0.6, 0.5)
	miR-20a-5p	(549, 289, 69, 16)	miR-142-3p	(41, 28, 1.8, 0.3)	miR-23b-3p	(14, 2.8, 1.3, 0.8)
	let-7b-5p	(381, 144, 69, 13)	miR-26b-5p	(39, 24, 1.3, 0.4)	miR-24-3p	(12, 3.6, 0.4, 0.3)
	miR-15a-5p	(304, 131, 23, 6.9)	miR-103a-3p	(39, 23, 1.4, 0.5)	miR-191-5p	(11, 9.1, 0.6, 0.3)
	miR-21-5p	(231, 101, 85, 45)	let-7a-5p	(38, 11, 3.7, 1.1)	miR-424-5p	(11, 2.2, 3.3, 1.0)
	miR-19b-3p	(224, 135, 6.9, 2.1)	miR-124-3p	(30, 1.7, 0.3, 0.3)	miR-181a-5p	(10, 4.2, 0.5, 0.4)
	miR-223-3p	(192, 73, 40, 9.3)	miR-107	(29, 17, 1.0, 0.4)	miR-27a-3p	(10, 1.3, 0.2, 0.1)
High	miR-106a-5p	(167, 97, 18, 5.2)	miR-320a	(24, 21, 1.3, 0.5)	miR-29a-3p	(8.4, 0.7, 0.4, 0.3)
	miR-15b-5p	(147, 65, 21, 5.7)	miR-9-5p	(23, 1.0, 0.4, 0.3)	let-7d-5p	(8.0, 4.1, 0.9, 0.2)
	miR-25-3p	(120, 142, 2.0, 1.1)	miR-23a-3p	(22, 8.4, 7.0, 3.4)	miR-29b-3p	(7.7, 1.4, 0.3, 0.1)
	miR-101-3p	(109, 64, 12, 2.9)	miR-27b-3p	(20, 1.5, 0.3, 0.2)	miR-126-3p	(7.6, 11, 0.1, 0.04)
	miR-486-5p	(107, 232, 2.7, 0.8)	let-7f-5p	(18, 8.3, 2.2, 0.6)	miR-204-5p	(7.1, 6.3, 4.9, 2.3)
	miR-92a-3p	(95, 213, 3.8, 1.9)	miR-29c-3p	(18, 3.3, 1.0, 0.4)	miR-18a-5p	(6.1, 3.8, 0.1, 0.02)
	miR-93-5p	(80, 49, 1.8, 0.6)	let-7c-5p	(16, 1.4, 0.6, 0.2)	miR-425-5p	(5.2, 6.0, 0.2, 0.1)
	miR-106b-5p	(77, 39, 4.0, 1.2)	miR-148b-3p	(16, 5.1, 1.7, 0.6)	miR-26a-5p	(4.8, 3.5, 0.6, 0.3)
	let-7i-5p	(67, 35, 4.8, 1.5)	miR-32-5p	(15, 8.0, 0.7, 0.3)	miR-194-5p	(4.1, 5.4, 0.1, 0.04)
	let-7e-5p	(4.1, 1.1, 0.5, 0.2)	miR-22-3p	(1.7, 1.4, 0.1, 0.04)	miR-146a-5p	(0.8, 0.5, 0.3, 0.2)
	miR-132-3p	(4.0, 0.5, 0.2, 0.2)	miR-222-3p	(1.7, 1.5, 0.2, 0.1)	miR-30a-5p	(0.8, 0.2, 0.02, 0.01)
	miR-192-5p	(3.9, 3.5, 0.2, 0.1)	miR-143-3p	(1.6, 0.5, 1.1, 0.3)	miR-34a-5p	(0.7, 0.1, 0.1, 0.1)
	miR-142-5p	(3.9, 2.6, 0.7, 0.3)	miR-186-5p	(1.5, 1.0, 0.1, 0.1)	miR-138-5p	(0.7, 0.04, 0.01, 0.01)
	miR-652-3p	(3.8, 2.2, 0.5, 0.1)	miR-30c-5p	(1.4, 2.5, 0.2, 0.1)	miR-378a-3p	(0.7, 0.5, 0.1, 0.1)
Moderate	miR-342-3p	(3.1, 1.0, 0.4, 0.2)	miR-99b-5p	(1.2, 0.1, 0.03, 0.04)	miR-146b-5p	(0.7, 0.4, 0.03, 0.02)
	miR-140-3p	(3.1, 2.3, 0.1, 0. 1)	miR-125a-5p	(1.2, 0.2, 0.04, 0.1)	miR-30e-5p	(0.7, 1.0, 0.1, 0.03)
	miR-150-5p	(2.1, 2.0, 1.0, 0.6)	miR-128-3p	(1.1, 0.6, 0.02, 0.03)	miR-145-5p	(0.4, 0.2, 0.03, 0.02)
	miR-181b-5p	(2.0, 0.2, 0.1, 0.1)	miR-30b-5p	(1.0, 1.6, 0.1, 0.1)	miR-92b-3p	(0.3, 0.3, 0.01, 0.01)
	miR-590-5p	(1.8, 1.0, 0.1, 0.03)	miR-532-5p	(0.9, 0.7, 0.03, 0.01)		
	miR-197-3p	(0.06, 0.08, 0.02, 0.01)	miR-155-5p	(0.01, 0.02, 0.01, ND)	miR-182-5p	(0.01, 0.01, ND, ND)
Low	miR-181c-5p	(0.03, 0.01, ND, ND)				

d days
ND Not Detected
