# Supplementary material for: Real-Time PCR Quantification of 87 miRNAs from Cerebrospinal Fluid: miRNA Dynamics and Association with Extracellular Vesicles after Severe Traumatic Brain Injury
Source: Int J Mol Sci. 2023 Mar 1;24(5):4751. doi: 10.3390/ijms24054751 (PMC10003046; doi:10.3390/ijms24054751)
Supplement: Supplementary file 1 [file ijms-24-04751-s001.zip › Table S2.pdf]

Table S2. Amounts of targeted microRNAs (miRNAs) in free proteins (FP) and extracellular vesicles (EVs) enriched fractions.

		**miRNA in fg**	**FP (d1-2, d3-4, d5-6, d7-12)<br>EV (d1-2, d3-4, d5-6, d7-12)**		
miR-451a	2211, 239.1, 20.76, 1.56<br>12.37, 4.28, 5.23, 0.63	miR-107	1.91, 0.27, <0.01, ND<br>0.07, 0.01, 0.02, ND	miR-140-3p	0.26, 0.04, ND, ND<br>0.04, 0.01, 0.01, ND
miR-144-3p	161.8, 15.60, 1.47, 0.15<br>1.97, 1.64, 0.98, 0.09	**miR-125b-5p**	1.91, 0.05, 0.01, ND<br>0.46, 0.02, **0.07**, 0.01	miR-24-3p	0.24, 0.01, ND, ND<br>0.06, ND, 0.02, ND
miR-20a-5p	98.20, 12.83, 1.35, 0.16<br>0.37, 0.07, 0.11, 0.01	miR-29a-3p	1.86, 0.01, 0.01, ND<br>0.04, 0.01, 0.01, <0.01	miR-132-3p	0.24, ND, ND, ND<br>0.01, 0.01, <0.01, ND
let-7b-5p	42.22, 3.81, 0.54, 0.05<br>0.39, 0.01, 0.05, <0.01	**miR-142-3p**	1.69, 0.11, 0.01, ND<br>0.66, 0.08, **0.43**, 0.03	miR-194-5p	0.23, 0.08, ND, ND<br>0.01, ND, <0.01, ND
miR-106a-5p	32.35, 3.86, 0.36, 0.03<br>0.18, 0.03, 0.06, <0.01	miR-100-5p	1.62, 0.01, ND, ND<br>0.05, ND, 0.01, 0.01	**miR-191-5p**	0.23, 0.05, <0.01, ND<br>0.07, 0.01, **0.02**, ND
**miR-16-5p**	26.69, 3.2, 0.08, 0.01<br>2.30, 0.21, **0.58**, 0.03	**miR-99a-5p**	1.59, <0.01, ND, ND<br>0.16, 0.02, **0.04**, 0.01	miR-99b-5p	0.16, ND, ND, ND<br>0.01, ND, <0.01, ND
miR-106b-5p	15.19, 1.52, 0.04, <0.01<br>0.19, 0.05, 0.08, 0.01	**miR-92a-3p**	1.56, 2.46, 0.01, 0.01<br>0.38, 0.21, **0.17**, 0.02	miR-181b-5p	0.15, ND, ND, ND<br>0.04, 0.01, 0.01,<0.01
miR-101-3p	10.84, 0.70, 0.13, 0.01<br>0.14, 0.01, 0.04, 0.01	miR-23b-3p	1.51, 0.04, 0.02, <0.01<br>0.17, 0.01, 0.03, 0.01	miR-186-5p	0.11, 0.01, ND, ND<br>ND, ND, ND, ND
miR-93-5p	10.56, 1.43, 0.02, 0.01<br>0.11, 0.04, 0.04, 0.01	**miR-27b-3p**	1.43, 0.01, <0.01, ND<br>0.11, <0.01, **0.02**, ND	miR-532-5p	0.11, 0.01, ND, ND<br>0.01, ND, ND, ND
**miR-21-5p**	8.39, 0.39, 0.41, 0.01<br>0.39, 0.10, **0.42**, 0.05	miR-29b-3p	1.30, 0.01, 0.01, ND<br>0.01, ND, ND, ND	miR-146a-5p	0.08, <0.01, ND, ND<br>0.01, ND, <0.01, ND
let-7i-5p	7.72, 0.75, 0.03, <0.01<br>0.06, 0.03, 0.03, 0.01	miR-424-5p	1.28, 0.04, 0.02, ND<br>0.01, ND, 0.01, ND	**miR-22-3p**	0.07, 0.01, <0.01, ND<br>0.04, **0.07**, **0.11**, 0.02
miR-15a-5p	7.21, 0.45, 0.01, <0.01<br>0.18, 0.06, 0.06, 0.01	**miR-486-5p**	1.23, 1.69, 0.01, ND<br>0.23, 0.09, **0.07**, 0.01	miR-590-5p	0.06, ND, ND, ND<br>0.01, ND, ND, ND
**miR-338-3p**	6.32, 0.02, 0.01, ND<br>0.49, 0.07, **0.16**, 0.03	**let-7c-5p**	1.22, 0.03, <0.01, ND<br>0.13, <0.01, **0.02**, ND	miR-342-3p	0.05, ND, ND, ND<br>0.02, ND, 0.01, ND
**miR-124-3p**	4.69, 0.04, 0.03, 0.03<br>0.07, 0.01, **0.02**, ND	miR-148b-3p	1.12, 0.08, 0.01, ND<br>0.02, <0.01, 0.01, ND	miR-222-3p	0.04, 0.01, ND, ND<br><0.01, ND, <0.01, ND
miR-29c-3p	4.49, 0.05, 0.02, ND<br>0.07, 0.04, 0.02, 0.01	**miR-204-5p**	1.06, 0.01, 0.03, ND<br>**0.98**, **0.32**, **1.05**, 0.22	miR-146b-5p	0.04, <0.01, ND, ND<br>0.01, ND, <0.01, ND
let-7g-5p	3.88, 0.51, 0.01, ND<br>0.14, 0.01, 0.04, 0.01	miR-148a-3p	1.03, 0.12, 0.01, ND<br>0.01, <0.01, 0.01, ND	miR-378a-3p	0.04, ND, ND, ND<br>0.01, ND, <0.01, ND
let-7a-5p	3.72, 0.29, 0.02, ND<br>0.41, 0.01, 0.10, <0.01	let-7d-5p	1.02, 0.08, <0.01, ND<br>0.05, <0.01, 0.02, ND	miR-138-5p	0.04, ND, ND, ND<br>0.01, ND, <0.01, ND
miR-320a	3.63, 0.57, 0.02, <0.01<br>0.26, 0.05, 0.10, 0.01	miR-142-5p	0.96, 0.08, 0.01, ND<br>0.04, 0.01, 0.04, ND	miR-34a-5p	0.04, ND, ND, ND<br><0.01, ND, ND, ND
miR-9-5p	3.24, 0.01, <0.01, ND<br>0.18, 0.01, 0.01, 0.01	miR-18a-5p	0.87, 0.09, ND, ND<br>0.01, 0.01, 0.01, ND	miR-150-5p	0.03, 0.01, ND, ND<br>0.02, 0.01, 0.03, 0.01
miR-23a-3p	3.13, 0.15, 0.10, 0.02<br>0.12, 0.01, 0.06, 0.01	miR-30c-5p	0.95, 0.04, 0.06, 0.01<br>**1.36**, 0.03, 0.09, 0.05	miR-128-3p	0.02, 0.01, ND, ND<br>0.01, ND, <0.01, ND
**miR-223-3p**	3.10, 0.09, 0.03, <0.01<br>0.75, 0.07, **0.71**, **0.06**	**miR-27a-3p**	0.79, 0.01, <0.01, ND<br>0.09, 0.01, **0.03**, 0.01	miR-30a-5p	0.02, ND, ND, ND<br>0.11, ND, <0.01, ND
miR-15b-5p	3.09, 0.17, 0.01, ND<br>0.11, 0.01, 0.04, <0.01	miR-652-3p	0.72, 0.06, <0.01, ND<br>0.01, ND, 0.01, ND	**miR-30b-5p**	0.01, 0.01, ND, ND<br>**0.09**, 0.01, 0.03, ND
**miR-103a-3p**	3.03, 0.31, <0.01, ND<br>0.14, 0.01, **0.04**, 0.01	miR-181a-5p	0.69, 0.04, ND, ND<br>0.28, 0.03, 0.06, 0.01	miR-30e-5p	0.01, ND, ND, ND<br>0.02, ND, 0.01, <0.01
**miR-19b-3p**	2.72, 0.13, 0.02, ND<br>0.28, 0.08, **0.11**, 0.01	miR-32-5p	0.56, 0.04, ND, ND<br>0.02, <0.01, 0.01, ND	miR-145-5p	0.01, ND, ND, ND<br>0.02, ND, 0.01, ND
let-7f-5p	2.72, 0.29, 0.01, ND<br>0.21, 0.01, 0.04, ND	miR-126-3p	0.48, 0.08, ND, ND<br>0.08, ND, 0.01, ND	**miR-92b-3p**	0.01, <0.01, ND, ND<br>**0.03**, ND, <0.01, ND
miR-26b-5p	2.62, 0.39, 0.01, <0.01<br>0.09, 0.01, 0.03, 0.01	let-7e-5p	0.39, 0.01, <0.01, ND<br>0.02, ND, 0.01, ND	miR-181c-5p	<0.01, ND, ND, ND<br>ND, ND, ND, ND
**miR-25-3p**	2.33, 0.38, 0.01, ND<br>0.14, 0.03, **0.04**, 0.01	miR-26a-5p	0.31, 0.04, ND, ND<br>0.14, <0.01, 0.03, <0.01	miR-125a-5p	0.01, ND, ND, ND<br>0.03, 0.01, 0.01, ND
		miR-425-5p	0.30, 0.10, ND, ND<br>0.02, 0.01, 0.01, <0.01	miR-197-3p	0.01, ND, ND, ND<br>0.01, ND, <0.01, ND
		miR-143-3p	0.29, <0.01, 0.01, ND<br>0.01, ND, <0.01, ND	miR-155-5p	ND, ND, ND, ND<br>ND, ND, ND, ND
		miR-192-5p	0.27, 0.03, <0.01, ND<br>0.01, 0.01, <0.01, ND	miR-182-5p	ND, ND, ND, ND<br>ND, ND, ND, ND
